# Complete Genome Sequence of the Plant Pathogen Ralstonia solanacearum Strain CIAT-078, Isolated in Colombia, Obtained Using Oxford Nanopore Technology

**DOI:** 10.1128/MRA.00448-20

**Published:** 2020-05-28

**Authors:** Diana López-Alvarez, Ana M. Leiva, Israel Barrantes, Juan M. Pardo, Viviana Dominguez, Wilmer J. Cuellar

**Affiliations:** a International Center for Tropical Agriculture (CIAT), Cali, Colombia; b Faculty of Agricultural Sciences, National University of Colombia, Palmira, Colombia; c Institute for Biostatistics and Informatics in Medicine and Ageing Research, Rostock, Germany; Broad Institute

## Abstract

Moko is one of the main diseases affecting banana and plantain in Colombia. Here, we report the genome sequence of the causal agent, the bacterium Ralstonia solanacearum (Smith) strain CIAT-078, collected in 2004 from affected plantains in central-west Colombia. The assembled genome was obtained using Oxford Nanopore Technology.

## ANNOUNCEMENT

Ralstonia solanacearum (Smith) belongs to a species complex of soilborne phytopathogenic bacteria that colonize the xylem tissue of hundreds of plant species worldwide (1). It is classified using molecular and biological methods into three species, four phylotypes, and several races; isolates belonging to phylotype II and race 2 cause Moko disease in banana and plantains (2, 3). Moko has been observed in Colombia since 1954 (4); the oldest leaves of affected plants show yellowing and wilting, become necrotic, and eventually collapse. Most importantly, the fruit pulp becomes discolored, causing major commercial losses (5, 6).

For genome sequencing, we selected CIAT-078, a pathogenic strain collected in 2004 from Moko-affected fields (Quindio, Colombia) that was previously characterized at the pathogenicity and biochemical level (7, 8) and used in routine screening for resistance assays (6). CIAT-078 was reactivated in semiselective medium South Africa (SMSA) (9) for 4 days and then in nutrient agar (Difco, USA) for 48 hours at 28°C. The bacteria were grown in LB medium prior to DNA extraction (Puregene Yeast/Bact. kit; Qiagen), and pathogenicity was confirmed by infecting ‘Dominico Harton’ plantains (6). Libraries were prepared from 1 μg of DNA using ligation sequencing kit 1D (catalog number SQK-LSK109) and sequenced using R9.4 chemistry (FLO-MIN106D) (10). Default settings were used for all software unless specified. Raw signals were base called using Guppy v3.4.3, and a total of 291,746 raw reads (*N*_50_, 4.27 kb) were generated. An assembly of quality-controlled reads (fastq pass) was done with Minimap2 (11) and Racon v1.4.7 (12) using strain UW163 as the reference (GenBank accession numbers NZ_CP012939 and NZ_CP012940). UW163 was selected due to the quality of its assembly, taxonomic classification (phylotype II), and host plant (plantain) (13). A second assembly was carried out using the UW163-based assembly as a reference. Assembly metrics were calculated with Qualimap v2.2 (14), and the genome was annotated using the Prokaryotic Genome Annotation Pipeline (15).

The circular chromosome of CIAT-078 consists of 3,481,951 base pairs (bp) (G+C content of 66.6%; 70× coverage). It contains 3,238 protein-coding sequences, 50 tRNA genes, 7 rRNA genes, 2 clustered regularly interspaced short palindromic repeat (CRISPR) loci, 5 riboswitches, 3 noncoding RNAs, 1 transfer-messenger RNA gene, and 4 AL1L pseudoknots. The circular megaplasmid consists of 1,907,373 bp (G+C content of 66.8%; 59× coverage) and contains 1,543 protein-coding sequences, 1 tRNA gene, and 2 riboswitches. The genes *rplB*, *mutS*, and *egl*, used to classify the bacteria at the phylotype and sequevar levels (2, 3), were located at positions 1381053 (*rpl*) and 2635266 (*mutS*) of the chromosome and position 1808381 (*egl*) of the megaplasmid. A maximum likelihood phylogeny of these genes grouped CIAT-078 within phylotype IIB sequevar 4 isolates, a subgroup representative of central-west Colombia (16). DUF3313, a recently identified sequence for diagnostics (6), was located at position 3277102 of the chromosome. The complete genome sequence of CIAT-078 will contribute to comparative genome and functional studies.

### Data availability.

The genome sequence of CIAT-078 was deposited in GenBank under accession numbers CP051295 and CP051296. Raw reads were deposited under SRA accession number SRP250670 (BioProject number PRJNA608676).
